# Identifying and Preserving Parathyroid Glands During Thyroid Surgery Using Indocyanine Green and a Review of the Literature

**DOI:** 10.1002/cnr2.70226

**Published:** 2025-06-23

**Authors:** Maziar Motiee‐Langroudi, Athena Farahzadi, Mohammad Shirkhoda, Habibollah Mahmoodzadeh, Ramesh Omranipour, Amirmohsen Jalaeefar, Iraj Harirchi

**Affiliations:** ^1^ Head and Neck Surgeon Imam Khomeini Complex Hospital, Cancer Institute, Tehran University of Medical Sciences Tehran Iran; ^2^ Division of Surgical Oncology Cancer Institute, Tehran University of Medical Sciences Tehran Iran; ^3^ Department of General Surgery Tehran University of Medical Sciences, Cancer Institute Tehran Iran

**Keywords:** hypoparathyroidism, indocyanine green, near infrared fluorescence imaging, near‐infrared fluorescence imaging, parathyroid gland, systematic review, thyroid surgery

## Abstract

**Background:**

The research aims to investigate the potential use of near‐infrared fluorescence imaging with Indocyanine green (ICG) to detect the parathyroid glands (PGs) during surgery and to evaluate their blood supply. Additionally, a review of existing literature was performed to evaluate the utility of near‐infrared imaging with ICG in identifying parathyroid glands and reducing the incidence of postoperative hypoparathyroidism, a common complication associated with this type of surgery.

**Methods:**

The study was carried out at the Cancer Institute, Tehran University of Medical Science, from December 2022 to April 2023, with 30 subjects participating in the research. In order to identify the PGs and evaluate their vascularization after resection, the patients were given an injection of 5 mg of Indocyanine green (ICG). To determine the usefulness of this technique, a comprehensive review of existing research was conducted using the PubMed and Cochrane Library electronic databases for relevant studies published up to September 2023. The reference lists within those studies were also examined to capture any further relevant research.

**Result:**

A total of 30 surgical procedures were carried out, during which the calcium level of the patients was monitored. On the first day post‐surgery, calcium levels ranged from 7 to 9.8 mg/dL, with an average of 8.4 mg/dL, and a median (interquartile range) of 8.5 mg/dL (7.87–8.82 mg/dL). On the tenth day post‐surgery, the mean calcium level was 8.34 mg/dL, with a median (interquartile range) of 8.2 mg/dL (8–8.6 mg/dL). Out of all the patients, 23.6% had avascular parathyroid glands and underwent auto transplantation. In two patients, the surgical team was only able to identify the parathyroid gland (PG) with the help of Indocyanine green (ICG); for the remaining 28 patients, this technique confirmed the presence of parathyroid tissue. A total of 72 PGs were detected with near‐infrared fluorescence imaging (NIRF) imaging. There were no complications during or after surgery due to the use of ICG. This article reported on 34 studies where a total of 1699 procedures were conducted, which included total thyroidectomy, lobectomy, and parathyroidectomy. The average dose of ICG administered was found to be 6.96 mg, with a median (interquartile range) of 7.5 mg/dL (5–10 mg/dL). Our findings showed that ICG successfully helped surgeons locate parathyroid glands in an average of 88.25% of cases.

**Conclusion:**

Our research indicates that utilizing Indocyanine green with a fluorescence imaging system is a safe, straightforward, and efficient method to assist surgeons in avoiding injury to the parathyroid glands during surgery and potentially anticipating complications such as post‐operative hypoparathyroidism. However, it should not be relied upon as the sole screening strategy. It is better to be used after surgeons have initially identified the parathyroid glands.

## 1 Introduction

Frequent complications following total thyroidectomy include hypoparathyroidism or hypocalcemia. Approximately 27% of patients undergo temporary hypoparathyroidism, while about 1% experience a permanent form. If a patient experiences transient hypoparathyroidism, it may increase their length of stay in the hospital and overall costs. On the other hand, permanent hypoparathyroidism may lead patients to take medication for the rest of their lives, which can significantly impact their quality of life [1], it's crucial for surgeons to locate and protect the parathyroid glands during thyroid surgery. Finding and assessing parathyroid glands during surgery traditionally depends heavily on the surgeon's skill and visual examination, which can be impacted by factors such as bleeding during surgery and ectopic parathyroid glands [1] There are two invasive alternatives for identifying during surgery: The frozen section analysis involves sacrificing a PG slice, while the “float or sink” method requires PG auto transplantation based on tissue density [2, 3].

Other techniques used to locate parathyroid glands include the use of methylene blue, dynamic photodetection by 5‐aminolevulinic acid (5‐ALA), and intraoperative Parathyroid hormone assay (IOPTH). There's also a newer method called the immune colloidal gold technique. This technique works by exploiting the difference in parathyroid hormone levels between parathyroid glands and other tissues. While it can quickly identify these glands during surgery (around 6 min), it's important to note that this method can sometimes mistake other tissues for parathyroid glands (false positive rate of 20%) [2, 4, 5].

A recent advancement in thyroid and parathyroid surgery is near‐infrared auto fluorescence imaging (NIAFI), which helps surgeons better visualize these glands during surgery. This method utilizes the auto fluorescence (AF) of parathyroid glands. The discovery of parathyroid glands' auto fluorescence paved the way for its use in surgery. While the initial finding of this property dates back to 2006 (Das et al.), it was Paras et al. in 2011 who utilized near‐infrared spectroscopy to identify these glands during surgery [1, 2, 6, 7] They found that parathyroid glands naturally glow more brightly than surrounding tissue when exposed to near‐infrared light, which allows for more accurate identification in real time. Further studies have shown that AF can improve intraoperative identification of parathyroid glands and decrease the number of patients experiencing low parathyroid hormone levels after surgery. McWade et al. validated these findings and demonstrated that using near‐infrared auto fluorescence (NIRAF) to identify parathyroid glands boasts an impressive 97% accuracy rate overall [1, 2, 8].

While NIRAF excels at identifying parathyroid glands, it doesn't provide information on how well these glands are receiving blood. This limitation means NIRAF alone may not be sufficient to decide if a gland needs to be transplanted elsewhere in the body. However, Indocyanine green (ICG) fluorescence imaging can effectively address this issue. ICG is a safe fluorescent dye that got its first clinical approval in 1956 [7]. It has a fast metabolism and minimal adverse effects. After intravenous injection, it quickly binds with plasma proteins and is a well‐established tool in angiography, used across various surgeries to visualize blood flow. ICG doesn't selectively bind to one tissue. Instead, it illuminates tissues with high blood flow more intensely, such as parathyroid, making it useful for defining the borders, functions, and viability of organs or tissues [9]. This method was first explored in a canine model in 2015, using intravenous administration of ICG to visualize the parathyroid glands [10, 11].

Several research studies have investigated the use of ICG fluorescence angiography in detecting parathyroid glands (PGs) and evaluating their health during surgeries to remove most or all of the thyroid or parathyroid glands [12, 13, 14]. However, there are limitations to using ICG in identifying parathyroid glands during surgery. The studies varied in their design, participant demographics, definition of success, and patient treatment methods. There is no universally agreed‐upon procedure or standard for assessing the results, making it challenging to conclusively link surgical findings to patient outcomes [15]. For instance, while Yin et al. found a strong association between well‐perfused parathyroid glands (ICG score ≥ 2) and normal postoperative PTH levels, supporting Fortuny et al.'s findings, Rudin et al. reported a need for at least two vascularized glands for normal function and questioned the reliability of ICGF due to its accuracy and sensitivity limitations. This discrepancy may be attributed to the subjective nature of ICG score evaluation. Lang et al. demonstrated that quantitative fluorescence intensity analysis, such as the use of the index of greatest and average Fluorescent Index (FI) to predict hypocalcemia risk, could offer a more objective and accurate assessment [1].

In this study, we assessed the effectiveness of ICG in detecting PGs, evaluating their vascularity, and assessing early postoperative hypoparathyroidism and hypocalcemia in 30 patients who had a total thyroidectomy, or lobectomy with or without cervical lymphadenectomy, for the first time in our country. Furthermore, we conducted a pooled analysis of existing literature on the usefulness of this technique and did a systematic review.

## 2 Materials and Methods

### 2.1 Patients

The research was carried out at the Cancer Institute at Tehran University of Medical Sciences between December 2022 and April 2023. The medical ethics committee approved the study, which was conducted adhering to the ethical guidelines set forth in the Declaration of Helsinki (2014 update). The ethical code registration for the study is IR.TUMS.IKHC.REC.1401.046.

The study involved adult patients (18 years or older) of both genders who were scheduled for elective hemi thyroidectomy or total thyroidectomy with or without cervical lymphadenectomy, who had normal liver and kidney function and provided written informed consent. Subjects with known hypersensitivity to ICG, iodine, penicillin, sulfa, pregnancy, lactation, and those who had received an injection of heparin within the past 24 h were excluded (all contraindications for intravenous ICG use). A fluorescence imaging system (Stryker 1588) and ICG dyes were used to identify the parathyroid glands in all participants during the surgical procedure, which included 30 patients.

### 2.2 Search Method to Find Relevant Research on This Topic

The authors looked for articles in two major medical databases, PubMed and Cochrane, between August and September 2023. The search was conducted using the following terms ((Indocyanine green) OR ICG) AND intraoperative AND ((thyroid) OR parathyroid).

We started by reviewing the titles and summaries of articles. If an article seemed relevant, then we retrieved the full text for a closer look. Additionally, we searched the bibliographies of these in‐depth articles for other potentially useful studies. The authors excluded editorials, letters to the editor, and articles describing a single doctor's personal technique. One researcher (A.F.) was responsible for reviewing all the retrieved articles based on their titles, abstracts, and full content.

The researchers included studies that met specific requirements. First, the study had to report results on using ICG during surgery. Additionally, the study had to address at least four out of these five points:

1. Research questions: Focused on using ICG during surgery for the thyroid or parathyroid glands.
2. ICG dosage: Specified the amount of ICG dye used (either a single amount or a range).
3. When ICG was given: Explained at what point during surgery the ICG dye was administered.
4. Blood calcium and PTH levels: Provided data on patients' blood calcium and parathyroid hormone (PTH) levels after surgery. This data could cover short‐term, mid‐term, or long‐term follow‐up.
5. The presence or absence of complications.

Exclusion criteria were: (1) Surgeons only used ICG after removing the thyroid gland to identify patients at risk of low calcium levels after surgery. (2) studies that were not available in English. (3) studies performed on animals.

### 2.3 Surgical Procedure and Indocyanine Green Angiography Protocol

Patients were given general anesthesia with endotracheal intubation. They were positioned supine with their neck slightly extended. The surgeon made a 4–6 cm incision at the suprasternal notch. Next, the subplatysmal layer was carefully dissected from the suprasternal notch to the level of the thyroid cartilage. After transection of the mylohyoid muscles in the midline, the surgeon used an ultrasonic knife to carefully dissect around the thyroid gland.

Surgical exploration for parathyroid glands begins with meticulous dissection to expose the thyroid lobes, prioritizing identification and protection of the recurrent laryngeal nerve (RLN). Subsequently, dissection proceeds along the posterior thyroid aspect, utilizing the inferior thyroid artery (ITA) as a key landmark, as parathyroid glands typically receive blood supply from its branches. A systematic visual and tactile search is conducted, recognizing the glands' characteristic yellowish‐brown color and ovoid shape, in common locations such as near the RLN‐ITA junction for superior glands, the lower thyroid pole or thyrothymic tract for inferior glands, and maintaining vigilance for ectopic locations like thyroid tissue itself. Meticulous dissection and preservation of surrounding tissues are paramount to prevent inadvertent removal or damage to the parathyroid glands and their blood supply.

After elevation of one thyroid lobe, ICG angiography was performed to help locate the parathyroid glands or confirm the assumed PGs and assess their blood flow. The same procedure was performed on the contralateral lobe in total thyroidectomy. After resection, the surgeon carefully examined the removed thyroid tissue to ensure no parathyroid glands were accidentally included before sending the tissue samples for further analysis under a microscope.

During the surgical procedure, the ICG dye was prepared by mixing 25 mg with 10 mL of sterile water. Following adequate exposure and elevation of each thyroid lobe, anesthesiologists injected 5 mg (2 mL) of this solution intravenously during surgery. The parathyroid glands absorbed the dye and were visualized. The injection could be repeated if needed, but the total amount used could not exceed 25 mg per patient, and the maximum dose used in the study was 20 mg. We used a laparoscopic fluorescence imaging system (Stryker 1588AIM camera system) for visualization of PGs. We assumed that all glands were either clearly devascularized or well perfused.

For parathyroid glands that appeared well‐vascularized by naked eye examination but ICG angiography revealed poor blood flow, the surgeons performed a parathyroid autotransplantation. In these instances, and where a parathyroid gland is deemed at risk of irreversible damage or has been inadvertently excised during surgery, autotransplantation is performed to preserve parathyroid function. The compromised gland is meticulously excised and immediately placed in a sterile, ice‐cold saline solution to minimize ischemic injury and maintain cellular viability. The excised tissue is then carefully dissected to remove all extraneous adipose and connective tissue, isolating the functional parathyroid parenchyma. This purified tissue is subsequently minced into uniform fragments, typically measuring 1–2 mm in diameter, to maximize the surface area available for revascularization and enhance graft survival. Small, precisely created pockets or slits are formed within the muscle fibers of the sternocleidomastoid muscle, ensuring minimal trauma to the surrounding tissue. The minced parathyroid fragments are then evenly distributed and implanted into these prepared pockets, avoiding excessive clumping to facilitate optimal vascular ingrowth. The muscle layers are carefully approximated and closed.

On the first and 10th day after surgery, all participants had blood drawn to measure calcium and parathyroid hormone (PTH) levels. These tests were performed in the hospital's clinical laboratories. As per the protocol, participants with severe hypocalcemia (less than 8 mg/dL) in the first 7 days after the operation were given oral calcium and vitamin D supplements. None of the 30 participants experienced severe permanent hypocalcemia in the 6 months following the operation. In total thyroidectomies, surgeons search for four parathyroid glands (PGs), while in hemi‐thyroidectomies, they search for two PGs.

### 2.4 Data Collection

The surgeons recorded several details during surgery: whether they were able to find the parathyroid glands using ICG fluorescence angiography, the total number of parathyroid glands identified using ICG, the number of parathyroid glands that showed poor blood flow based on ICG angiography, autotransplantation of PGs, the occurrence of intraoperative complications, total operating time, additional operation time for using ICG, ICG dose in mg, time of visualization, Ca level POD1, Ca level POD 10, PTH level POD1, need for intravenous Ca therapy, need for oral Ca therapy, history of hypothyroidism or hyperthyroidism, preoperative Ca level, pathology, adverse reaction to ICG, finding of PG in pathology, PG greatest dimension (cm), and preoperative vitamin D.

### 2.5 Data Abstraction

We have collected the following information for each study: (1) Study basics: This included the lead author's name, year of publication, type of study, and total number of patients involved. We also noted the specific imaging technique used and any special instruments mentioned. (2) Parathyroid gland details: We looked at how many parathyroid glands were found using the optical technique, how many were accidentally removed during surgery, and how many needed transplantation. (3) Calcium level follow‐up: We analyzed the percentage of patients who experienced low blood calcium levels at different time points after surgery. Low calcium was defined as a level below 8.0 mg/dL on the first or second day after surgery (short‐term), within 2 weeks (medium‐term), and after 6 months (long‐term) 0.4. PTH level follow‐up: We also examined the percentage of patients who developed low parathyroid hormone levels at different time points after surgery. Low PTH was defined as a level below 15 pg/mL on the first or second day after surgery (short‐term), within 2 weeks (medium‐term), and after 6 months (long‐term).

### 2.6 Statistical Analysis

The demographic parameters were summarized using descriptive statistics in this study. The study's findings were presented as averages (means/median) with standard deviations (SD)/quartile to show variability. Statistical analysis was performed using SPSS version 22.

## 3 Results

A total of 30 operations were performed on 30 patients, including lobectomy and total thyroidectomy with or without central and/or lateral neck dissection. Most patients were female (21 patients, 70%), and the main reason for thyroid surgery was papillary thyroid carcinoma (53.3%). The study participants had an average age of 43.93 years (SD =11.01). Further details of patient characteristics are shown in Tables 1 and 2. The calcium level on postoperative day 1 (POD 1) was a minimum of 7 mg/dL and a maximum of 9.8 mg/dL, with a mean of 8.4 mg/dL and a median (interquartile range) of 8.5 mg/dL (7.87–8.82 mg/dL). Mean Ca levels at day 10 were 8.34 mg/dL, with a median (interquartile range) of 8.2 mg/dL (8–8.6 mg/dL).

**TABLE 1:** Demographic parameters and tumor characteristics of the patients.

Gender		
Female	21	70%
Male	9	30%
Indication for surgery		
PTC	16	53.3%
MTC	1	3.3%
MNG	8	26.7%
Benign follicular nodular	3	10%
AUS	2	6.7%
Extent of surgery		
TT	15	50%
Lobectomy	4	13.3%
TT. central	6	20%
TT. MRND	5	16.7%
Need for IV Ca therapy		
No	23	76.7%
Yes	7	23.3%
Need for PO Ca therapy		
No	3	10%
Yes	27	90%
Number of avascular PG	17	23.6%
Number of evaluated parathyroid glands (*n* = 72)		
History of thyroid disorder		
Hyperthyroidism	2	6.75
Hypothyroidism	1	3.3%
Adverse reaction to ICG	None	
Inadvertent parothyroidectomy		
One PG	8	26.7%

Abbreviations: AUS, atypia of undetermined significance; Ca, calcium; ICG, Indocyanine green; IV, intravenous; MNG, multinodular goiter; MRND, modified radical neck dissection; MTC, medullary thyroid cancer; PG, parathyroid gland; PO, per oral; PTC, papillary thyroid cancer; TT, total thyroidectomy.

**TABLE 2:** Overview included patients and fluorescence imaging.

	Minimum	Maximum	Mean	SD	Median	25 percentile	75 percentile
Ca level POD1	7.0	9.8	8.400	0.7339	8.5	7.87	8.82
Ca level POD 10	7.7	9.7	8.347	0.4644	8.2	8	8.6
PTH level POD1	1.2	90.0	50.363	29.5578	58	20.32	80
Total number of PG identified by ICG in each patient	1	4	2.40	0.894	2	2	3
Time of operation (hour)	1.0	10.0	3.250	1.8651	3	2	4
Extra time add to surgery (minute)	60	600	236.00	110.222	5	4	7
Dose of ICG (mg)	2.5	20.0	8.500	3.3861	10	5	10
Time of visualization (second)	20	240	63.67	50.548	60	30	60
Preoperative ca	8.0	11.0	8.813	0.6213	8.8	8.5	9
Preoperative vitamin D	30	53	41.50	16.263	41.5	30	
PG greatest dimension (cm)	0.5	5.0	1.103	0.8410	1	0.5	1
PG found in specimen	0	1	0.27	0.450	0	0	1

Abbreviations: Ca, calcium; cm, centimeter; ICG, Indocyanine green; mg, milligram; N, number; PG, parathyroid gland; POD, post‐operative day; PTH, parathyroid hormone; SD, standard deviation.

Autotransplantation was performed on avascular PGs, which constituted 23.6% of all PGs. Postoperative day 1 (POD1) parathyroid hormone (PTH) levels exhibited significant variability, ranging from a minimum of 1.2 pg/mL to a maximum of 90.0 pg/mL. The median PTH level was 58 pg/mL, and the 25th and 75th percentiles were 20.32 pg/mL and 80 pg/mL, respectively. There were no complications during or after surgery due to the use of ICG. The average operation time was 190 min with a median (interquartile range) of 120 min (120–240 min). Near‐infrared fluorescence(NIRF) imaging with ICG resulted in an additional time (minute) of 5.57, and a median (interquartile range) of 5.25 min (4–7 min) during surgery.

**FIGURE 1:**
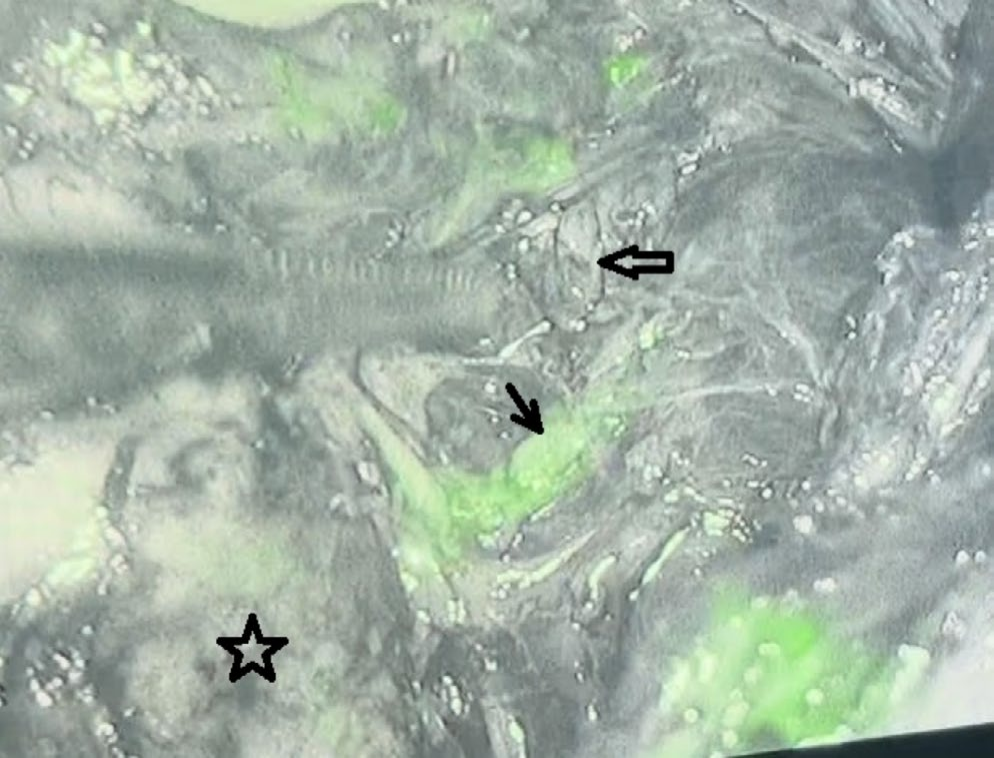
Near‐infrared fluorescence image from a 36‐year‐old male during total thyroidectomy for PTC, showing a well‐perfused (arrow) and avascular (empty arrow) parathyroid gland, alongside the left thyroid lobe (star). Diffuse background fluorescence is due to ICG distribution in the surgical field.

The application of indocyanine green (ICG) during surgery played a crucial role in identifying parathyroid glands (PGs) in two out of 30 patients. Without ICG, the surgical team might not have been able to locate these glands. In the remaining 28 patients, NIRF with indocyanine green was mainly used to confirm the presence of parathyroid tissue. A total of 72 PGs were detected with NIRF imaging. Figure 1, Video S1. At least one PG was found in each patient.

Examination of the surgical samples revealed one parathyroid tissue in eight patients, with six of them undergoing a total thyroidectomy. During the postoperative period, 23.3% of patients required IV calcium therapy and four experienced symptomatic hypocalcemia. None of the patients had a calcium level below 7 on the first postoperative day after surgery. In eight patients who had undergone total thyroidectomy, the calcium level temporarily fell below the normal range of our laboratory the following day after surgery. The calcium ion levels of the patients were 7, 7, 7.3, 7.9, 7.5, 7.8, 7.5, 7.8 mg/dL.

The number of identified parathyroid glands during surgery varied, with one gland found in 5 patients, two in 11, three in another 11, and four in the remaining 3. Notably, 24 patients, all of whom had at least one parathyroid gland with normal indocyanine green (ICG) angiography, exhibited postoperative PTH levels within the expected range. However, in six patients, where the ICG angiography showed at least one healthy blood flow to the parathyroid gland, temporary hypoparathyroidism developed on POD 1. None of the patients experienced permanent hypocalcemia.

**FIGURE 2:**
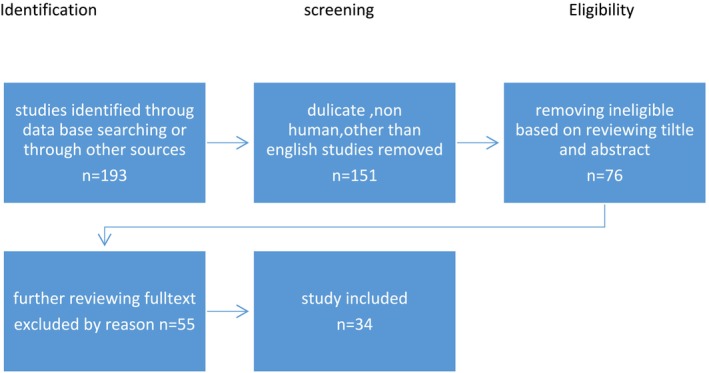
Flow diagram outlining information collection and selection of studies.

Our investigation began by identifying 193 relevant articles through a comprehensive search of PubMed (Medline) and the Cochrane Library, and additional articles were retrieved manually from the references section of the articles. Figure 2 illustrates the selection process in detail. After applying search limits, such as studies conducted on humans and manuscripts available in English, and eliminating duplicates, the number of records was reduced to 151. Initial screening based on title and abstract excluded 75 articles from further evaluation, while 76 were deemed potentially relevant. Upon further review, 42 articles were excluded. Following a rigorous selection process, 34 studies were chosen for further analysis. Table 3 provides a summary of their key characteristics.

**TABLE 3:** Characteristics of included studies.

	Authors/Year	Type/study design	Number	Surgery	Device	ICG dose (mg)	Detected PG	Increase in PG detection	Time of appearance (second)	Adverse effect	Pathology	Calcium	PTH
1	Jeffery M. Chakedis et al. 2015 [11]	Case report	1	Redo, parothyroidectomy	PINPOINT fluorescent imaging system (Novadaq)	7.5	100%		20	None	Recurrent HPT		
2	Sara Sound et al. 2015 [16]	Case report	3	Parothyroidectomy	Novadaq (Ontario, Canada)	9.15	100%		240	None			
3	Zaidi et al. 2016 [17]	Case series, prospective	33	Parothyroidectomy	PINPOINT	5	92.9%		30–60	None			
4	Zaidi et al. 2016 [18]	Case series	27	Total thyroidectomy	PINPOINT	5 mg before & 5 mg after TT	83.5%		60	None		11%	
5	Vidal Fortuny et al. 2016 [19]	Case series	36	Total thyroidectomy	PINPOINT	7.5–12.5	91.9%		60–120	None		0%	
6	Hyeong Won Yu et al. 2016 [20]	Case–control study	44control 22 ICG	BABA Robot thyroidectomy	Firefly system	10 (0.17 mg/kg)	97%		180	None	PTC		
7	Bora Kahramangil et al. 2017 [21]	Cohort, prospective	22	Total thyroidectomy, lobectomy	PINPOINT	5	95%			None		Early 4.5%	
8	Mohsin et al. 2017 [22]	Case report	1	Parothyroidectomy	N/A	N/A	100%			None			
9	Cui et al. 2017 [23]	Cohort, prospective	29 (with 20 w.o 9)	Parothyroidectomy	AISERY + J	0.5 mg/kg	91.1%		4500 ± 2100	None			
10	Lang et al. 2017 [24]	Case series	90 (70 with 4 PG identified)	Total thyroidectomy	SPY	2.5 mg after TT	95.3%		15	None		15.7%	
11	Alesina et al. 2018 [25]	Case series	5	Parothyroidectomy	Karl Storz Endoskope, Tuttlingen, Germany	7.5	100%		30–70	None	none	20%	20%
12	Vidal Fortuny et al. 2018 [26]	RCT	196	Total thyroidectomy	PINPOINT	Up to 5 mg/kg	77.6%		60–120	None		Early 0% medium/0% long 0%	5.55% 0% 0%
13	Jin et al. 2018 [27]	Case series	26	Total thyroidectomy	INS	5	100%		120	None		Early 0% Medium/0% long 0%	0% 0% 0%
14	van den Bos et al. 2018 [4]	Case series	26	Total thyroidectomy	Karl Storz GmbH & CO., Tuttlingen, Germany	15	43%		30	None		Early 11.5% medium 0% long 0%	0% 0% 0%
15	Jonathan C. DeLong et al. 2018 [10]	Cohort, retrospective	60	Parothyroidectomy	PINPOINT Endoscopic Fluorescence Imaging System (Novadaq Technologies Inc., Bonita Springs, FL)	7.5	93%		60	None			
16	Jin et al. 2019 [28]	Case series	3	Total thyroidectomy	INS	5	100%		120	None		0%/0%/0%	0%/0%/0%
17	Rudin et al. 2019 [29]	Cohort, retrospective (ICG: 86 patients; control: 124 patients)	210	Total thyroidectomy	PINPOINT camera	7.5	82%		60	None			37% me11.62% 3.12%
18	Max Lerchenberger et al. 2019 [30]	Cohort, prospective	50	Total thyroidectomy. parothyroidectomy	Storz NIR/ICG endoscopic system	5	81%		60–120	None			
19	Di Meo et al. 2019 [31]	Cohort, retrospective	37	Parothyroidectomy	PINPOINT camera	2.5	95.16%		30–60	None			
20	Shu‐Jia Peng et al. 2020 [32]	Case report	1	Lobectomy	Smart Eye 101	0.5 mg/kg	100%			None	PTC	nl	nl
21	Pablo Moreno Llorente et al. 2020	Cohort	50	Total thyroidectomy (W/WO central neck dissection)	SPY‐PHI	7.5 mg up to 5 mg/kg	81%		60–120	None		11%	
22	Theodosios S. Papavramidis et al. 2020 [33]	Cohort, prospective	60	Total thyroidectomy	OPAL1, Karl Storz SE & Co. KG, Tuttlingen, German	5	98.3%		120	None		26.66% early	11.66% early
23	Mehmet Ilker Turan et al. 2020 [9]	Cohort, retrospective	7	Trans oral endoscopic thyroidectomy and parothyroidectomy	Olympus VISERA ELITE 2 laparoscopic imaging system	10	100%		120	None		28.57% early	
24	Jared Matson et al. 2021 [34]	Cohort, retrospective	66	Parothyroidectomy	the SPY‐PHI	2.5	40%		26.7	None			
25	Parfentiev et al. 2021 [35]	RCT	58	Total thyroidectomy	Karl Storz, Tuttlingen, Germany	15			120	None		6.67%	
26	Liang et al. 2021 [36]	Cohort, retrospective	60	Trans oral endoscopic total thyroidectomy lobectomy	VISERA ELITE II system (Olympus, Tokyo, Japan)	7.5	85.4%	5.7%	60	None			
27	Pablo Moreno Llorente et al. 2022 [37]	Cohort, Prospective	94	Total thyroidectomy			88%			None		23.4%	
28	Quéré et al. 2022 [38]	Cohort, retrospective	32	Total thyroidectomy	The Novadaqsystem	5	60.15%	12%		None		37.5%	
29	Priyanka et al. 2022 [39]	Cohort, prospective	50	Total thyroidectomy			73%			None			
30	Volodymyr V. Grubnik et al. 2023 [40]	Cohort	92 (48 control 44ICG)	Total thyroidectomy						None		4.5%	
31	Shin‐Young Park et al. 2023 [41]	Cohort, retrospective	6	Robot‐assisted parothyroidectomy	The FireflyTM	10 (0.17 mg/kg)	100%		180	None			
32	Pablo Moreno‐Llorente et al. 2023 [42]	Cohort, prospective	120	Total thyroidectomy		10				None		5.6%	
33	Battistella E. et al. 2023 [43]	Cohort, retrospective	18	Parothyroidectomy		7.5	91.6%		60				
34	Garrett E. Rupp et al. 2023 [44]	Case report	1	Parothyroidectomy	The Stryker SPYPHI	2.5	100%		60	None			
35	Our study	Cohort, prospective	30	TT. lobectomy	Stryker 1588AIM camera system	8.5	64.28%	2.7%	63.67	None	PTC. MTC. MNG. AUS. benign follicular nodule	Early 05 Medium 0%	20%

Abbreviations: AUS, atypia of undetermined significance; HPT, hyper parathyroidism; ICG, indocyanine green; mg/kg, milligram/kg; MNG, multinodular goiter; MTC, medullary thyroid cancer; PG, parathyroid gland; PTC, papillary thyroid cancer; PTH, parathyroid hormone; RCT, randomized controlled trial; TT, total thyroidectomy.

Our review followed a systematic review format, focusing on presenting diverse surgical experiences. We opted not to include a meta‐analysis due to the potential for bias caused by small sample sizes in the included studies. Instead, we employed a narrative synthesis approach to identify and analyze relationships between studies using logical reasoning.

This article includes 34 studies conducted between 2015 and 2023 that used ICG angiography to identify and preserve the parathyroid gland and includes 18 cohort studies, 8 case series, 5 case reports, one case–control study, and two RCTs. A total of 1699 procedures were performed, including total thyroidectomy, lobectomy, and parathyroidectomy. The mean dose of ICG was found to be 6.96 mg, with a median (interquartile range) of 7.5 mg/dL (5–10 mg/dL). The mean detection rate for parathyroid glands with ICG was 88.25% with a median (interquartile range) of 93% mg/dL (82%–100%).

Chakedis et al. were the first to describe the use of ICG for parathyroid identification in one case in 2015 [11]. A study by Yu et al. investigated the use of indocyanine green angiography for parathyroid gland identification during robotic thyroidectomy with a bilateral axillo‐breast approach (BABA) and was able to locate it in 97% of cases in 2016 [20].

In 2018, Vidal Fortuny et al. published the first randomized controlled trial in this field. The study involved 196 patients undergoing a total thyroidectomy. The study successfully located parathyroid glands in 77.6% of the patients. The report showed no cases of early, medium, or late post‐operation hypocalcemia, but did report early post‐operation hypoparathyroidism in 5.55% of patients. However, there were no cases of medium or late post‐operation hypoparathyroidism [26].

In 2019, a study by Rudin et al. investigated the effectiveness of indocyanine green for preserving parathyroid glands during total thyroidectomy. The study included 210 patients, of which 86 received ICG angiography while 124 were in the control group. ICG angiography successfully identified the parathyroid glands in 82% of patients in the study. The study also reported that early, medium, and late postoperative hypoparathyroidism occurred in 37%, 11.62%, and 3.12% of patients in the ICG group [29].

Liang et al. reported a significant increase (5.7%) in the identification of parathyroid glands in patients undergoing Transoral Endoscopic Total Thyroidectomy and lobectomy with ICG compared to those who did not use ICG [36]. Similarly, Quéré et al. found a 12% improvement in parathyroid gland detection during total thyroidectomy when ICG angiography was used [38]. In the studies that were analyzed, no adverse effects were observed. The average time for the parathyroid gland to appear after ICG injection was found to be 255.83 s with a median of 75 s. If we exclude the Cui et al. 2017 study [23] from our analysis, the average time decreases to 86.06 s with a median (interquartile range) of 60 s (47.5–120 s). Notably, the Cui et al. study reported a significantly longer time of 4500 ± 2100 s to identify the parathyroid glands following ICG administration, which was much longer than the time reported in other studies.

The mean detection rate of parathyroid glands with ICG usage is 89%. However, we cannot draw a conclusion about the rate of postoperative hypocalcemia and hypoparathyroidism after using ICG, as most studies did not provide exact statistics on this issue. Out of 34 studies, only 18 reported data on postoperative hypocalcemia. Vidal Fortuny et al. [19, 26] and Jin et al. [27, 28] reported 0% postoperative hypocalcemia. On the other hand, Quéré et al. [38] reported 37.5% postoperative hypocalcemia in 32 patients. Out of 34 studies, 8 reported postoperative hypoparathyroidisms. Jin et al. [27, 28] and van den Bos et al. [4] reported 0% postoperative hypoparathyroidism. However, Rudin et al. [29] reported 37% of early postoperative hypoparathyroidism.

## 4 Discussion

Locating the parathyroid glands is essential during both thyroid and parathyroid surgery. However, its detection is often challenging due to the gland's varying number and location. In up to 31% of thyroidectomy cases, particularly those involving lymph node dissection of the pre‐ and paratracheal area as part of standard thyroid cancer surgery, unintended parathyroid gland removal or damage is experienced [13].

Our study observed a 20% incidence of transient postoperative hypoparathyroidism, with no cases of permanent hypoparathyroidism, aligning with the range of reported rates in the literature. For instance, Pastoricchio et al. found transient hypoPT in 12.5% of the Near‐Infrared (NIR) group and 19.2% of the No‐NIR group, while Yin et al. reported a significantly lower incidence in the Near‐Infrared Fluorescence Imaging (NIFI) group (27.8%) compared to the control group (43.3%, *p* = 0.029). Notably, Yin et al. further demonstrated that patients with well‐perfused parathyroid glands (PGs) in the NIFI group experienced a significantly reduced hypoparathyroidism rate (4.5%) compared to controls (34.6%, *p* < 0.001). These findings, alongside previous studies suggesting a transient hypoparathyroidism incidence of approximately 45%, highlight the variability in reported rates, potentially influenced by surgical technique, imaging modality, and patient selection [1, 5]. Our results fall within this spectrum, suggesting that while transient hypoparathyroidism remains a common postoperative complication, its incidence can vary significantly.

A surgeon's experience plays a critical role in minimizing the likelihood of hypoparathyroidism during surgery [1, 5]. Post‐operative hypoPT and hypocalcemia may require lifelong administration of large amounts of calcium drugs, which can be a complicated process that affects 5.5% of patients. Thus, it is important to detect the parathyroid gland during surgery and salvage it for auto transplantation. However, detecting the gland mainly depends on the surgeon's visual skills. Intraoperative frozen section analysis provides a rapid method for surgeons to confirm the identification of parathyroid glands during surgery. While some institutions are exploring real‐time measurements of intact parathyroid hormone (PTH) levels during surgery, this approach is not yet widely available. Even when available, PTH levels may not provide immediate enough feedback to definitively guide decisions about parathyroid gland preservation. Preoperative imaging techniques like ultrasound and CT scans can be helpful in identifying enlarged parathyroid glands, but they often struggle to detect glands of normal size. Similarly, while technetium sestamibi scans can aid in locating abnormal glands, they have limitations in reliably pinpointing healthy ones. This highlights the need for additional tools during surgery to ensure accurate parathyroid gland identification [13].

The need for a reliable method to locate parathyroid glands during surgery has been a major area of research since the pioneering work by Dudley in 1971 [7]. Dudley's initial approach involved using methylene blue as a contrast agent, but inconsistent results and potential toxicity, including serious neurological adverse effects, discouraged routine use of this technique. Despite exploring newer techniques like near‐infrared fluorescence imaging with lower doses of methylene blue and aminolevulinic acid delivered intravenously, these methods haven't achieved widespread adoption due to challenges encountered in practical use and their relatively low detection rate (around 45%) for parathyroid glands [45].

The real breakthrough in identifying parathyroid glands during surgery occurred in 2008. Researchers at Vanderbilt University identified a unique property of the parathyroid glands called auto fluorescence. This inherent property allows parathyroid tissue to glow upon exposure to near‐infrared light specifically at a wavelength of 785 nm. The emitted light has a wavelength between 820 to 830 nm and exhibits fluorescence 2–11 times higher than neighboring structures like the thyroid gland. This property facilitates the accurate detection and positioning of the parathyroid glands during surgical procedures. The exact molecule that causes this auto fluorescence is still unknown, but it is thought to be mediated by one of two receptors: the calcium‐sensing receptor or the vitamin D receptor, according to some experts [13, 46].

Since the discovery of this technique for detecting parathyroid glands during surgery, multiple research efforts have demonstrated the technique's high accuracy in detecting PGs, with a specificity exceeding 80% [47]. However, one downside of this technique is that differentiating thyroid and parathyroid tissue can be challenging due to the comparable intensity of their auto fluorescence in some cases like thyroiditis. Additionally, there is a risk of false positives due to overlapping auto fluorescence with other tissues. To address these limitations, a clinically approved fluorescent dye called indocyanine green (ICG) serves as a contrast agent to improve the differentiation of structures within the image. ICG allows for real‐time assessment of tissue perfusion and vascularization and has been used successfully in various procedures. The protocols and doses of ICG used may vary among different centers [45, 46].

ICG is not targeted specifically toward parathyroid parenchyma. The exact process by which parathyroid glands (PGs) accumulate indocyanine green (ICG) and achieve enhanced fluorescent differentiation from surrounding tissues remains unclear. However, one hypothesis suggests that the significantly higher blood flow in PGs compared to neighboring tissues might be a contributing factor [3].

Pioneering the use of ICG for NIRF imaging of parathyroid glands in animal models, Suh et al. were the first to investigate its effectiveness [3]. In 2015, Chakedis et al. reported the first case of hyperparathyroidism that underwent a redo parathyroidectomy with the help of ICG angiography [11]. Sound et al. further explored the use of NIRF imaging with ICG in a clinical setting. Their study involved three patients undergoing reoperative neck surgery for primary hyperthyroidism. Each patient received an initial dose of 5 mg ICG, with one patient receiving an additional 5 mg and two others receiving 3.25 mg. Notably, the parathyroid glands (PGs) became visible within 2 min of ICG administration in all three patients and remained fluorescent for up to 20 min [16]. Research by Yu et al. indicates that increasing the dose of ICG might lead to a stronger fluorescent signal [20]. In 2016, Hyeong Won Yu et al. conducted a case–control study on robotic Bilateral Axillo‐Breast Parathyroidectomy (BABA) for the first time and successfully identified parathyroid glands in 99.7% of cases in the ICG group [20]. Zaidi et al. published the first prospective study investigating the use of intraoperative ICG fluorescence imaging for parathyroid gland (PG) visualization during surgery for hyperparathyroidism (HyperPT). The study involved 33 patients [17].

In a 2016 study by Zaidi et al., researchers investigated the use of ICG for parathyroid gland visualization during surgery in 27 patients undergoing total thyroidectomy for various reasons (multinodular goiter, thyroid cancer, Graves' disease). They identified a total of 85 PGs, of which 71 (84%) displayed ICG uptake, indicating successful visualization. Interestingly, the parathyroid hormone (PTH) level measured on the first postoperative day correlated with the number of remaining PGs and the intensity of their fluorescence (*p* = 0.05), suggesting a potential link between ICG uptake and postoperative PTH levels [18].

In 2016, Vidal Fortuny et al. conducted a study on 36 patients who underwent ICG angiography during total thyroidectomy (TT). They described a two‐point grading system to assess the vascularity of the parathyroid glands. A score of 0 indicated no vascularization (no green color), 1 indicated partial vascularization (pale green color), and 2 indicated well‐vascularization (bright green color). Researchers identified one parathyroid gland (PG) in one patient, two PGs in 11 patients, three PGs in 18 patients, and four parathyroid glands (PGs) in 6 patients. However, ICG angiography failed to show a well‐vascularized PG in 6 patients, with two of them experiencing transient hypoparathyroidism. All patients with at least one well‐vascularized PG had normal postoperative PTH levels [19]. However, in our study, in six patients with healthy blood flow to the parathyroid gland, temporary hypoparathyroidism developed on POD 1, but none experienced permanent hypocalcemia.

In a 2017 study, researchers described a successful minimally invasive surgical procedure to remove a parathyroid gland tumor (adenoma) in a patient with hyperparathyroidism (HyperPT). The surgery used robotic technology (daVinciSi) and real‐time imaging with ICG to locate the tumor [22].

In 2018, Vidal Fortuny et al. conducted the first RCT and identified parathyroid glands in 77.6% of cases. The clinical study examined temporary postoperative hypoparathyroidism in 196 patients who received ICG angiography during thyroid surgery. Among the patients, 146 had at least one PG showing uptake of the imaging dye (ICG), indicating good function, while 50 did not. In total, 499 PGs were assessed, and over 77% (387) showed good blood flow with ICG. Interestingly, none of the patients with at least one well‐perfused PG developed hypoparathyroidism. However, 11 out of 50 patients who had no ICG uptake in any PG did experience this complication. The study suggests that ICG perfusion might predict which patients are unlikely to develop hypoparathyroidism after surgery, potentially reducing the need for routine calcium and parathyroid hormone (PTH) level checks after surgery in these patients [26].

In a study by Jin et al., researchers investigated the relationship between ICG angiography and parathyroid function after thyroid surgery in 26 patients with various thyroid conditions. The vascularity of each identified parathyroid gland was visually graded on a scale of 0 to 2 prior to ICG angiography. Following the procedure, the fluorescence intensity(FI) of each parathyroid gland was assessed using a 0–2 scale after ICG angiography, with 0, 1, and 2 representing weak, moderate, and strong fluorescence, respectively. The results showed that all 22 patients who had at least one PG with a strong ICG signal (score > 2) maintained normal levels of parathyroid hormone (PTH) after surgery. Conversely, two out of the four patients with a weak ICG signal (score < 2) in their PGs experienced temporary hypoparathyroidism [27].

DeLong et al. demonstrated the effectiveness of ICG fluorescence‐guided parathyroidectomy in their study. They found that out of 54 patients with a preoperative sestamibi scan, 18 had adenomas that could not be detected on pre‐operative sestamibi scans. However, all 18 of these adenomas were successfully located during surgery using ICG imaging. This finding emphasizes the value of ICG fluorescence during surgery, especially when traditional methods struggle to pinpoint the exact location of parathyroid glands. ICG's ability to detect these glands, even when they're missed before or during surgery visually, can be a significant advantage [10].

A study by McWade et al. identified several factors that can reduce the brightness of the imaging dye (ICG) used to assess parathyroid glands during surgery. These factors included high body mass index (BMI), cancerous tumors, vitamin D deficiency, and elevated calcium levels [48].

ICG angiography offers a significant advantage over previous methods by allowing analysis of individual parathyroid gland (PG) function. Before ICG, surgeons could only measure the combined function of all glands during surgery (intraoperative PTH assays). However, evaluating all four glands with ICG angiography is crucial for accurate prediction of overall parathyroid function. Another benefit is the ability to perform ICG angiography safely multiple times. However, the quality of the images progressively decreases with each injection. This is because the contrast between pre‐ and post‐injection phases weakens with repeated doses, depending on the time interval between them. As a result, subsequent near‐infrared (NIR) images may have reduced fluorescence contrast. Additionally, strong residual ICG fluorescence can even mask the natural NIR glow (NIRAF) of PGs, even after the first injection [49].

A systematic review and meta‐analysis of auto fluorescence and indocyanine green in thyroid surgery was conducted by Diego Barbiere and colleagues in 2021. The study included 13 papers with a total of 1484 procedures. The study found that NIFI techniques led to a noticeable decrease in the risk of low calcium levels after surgery, both in the short‐term and mid‐term following the procedure [49].

Our review identified a gap in the research on ICG angiography. While many studies focus on its effectiveness in locating parathyroid glands during surgery, there is a lack of data on its impact on short‐term, mid‐term, and long‐term calcium (hypocalcemia) and parathyroid hormone (PTH) levels after surgery. This makes it difficult for us to statistically analyze the impact of these crucial post‐surgical outcomes. In our study, 26.6% of patients had temporary hypocalcemia while no patients experienced permanent hypocalcemia.

It is worth noting that during surgery, both autofluorescence (AF) and the imaging technique using the dye ICG may not be sensitive enough to serve as screening tools for the localization of parathyroid tissue. This is because the parathyroid glands may be covered by fatty tissue that can obscure these imaging techniques, and even small hemorrhages in the surgical area can obscure the field [50, 51]. Although both AF and ICG imaging can confirm the presence of a parathyroid gland, they do not replace the accuracy of dissection by an experienced surgeon. Therefore, it is crucial to recognize the parathyroid glands with the naked eye beforehand. However, with the development of more advanced imaging systems and more accurate and objective assessment through quantitative evaluation of fluorescence intensity, effective screening at an early stage of surgery may become possible [52, 53, 54, 55].

We have come across 34 articles that are relevant to our research and include research where the parathyroid glands are found with the help of ICG, all of which are in English and include a total of 1699 procedures. The majority of these articles are observational studies, but a few randomized control trials have also been conducted by researchers such as Vidal Fortuny [26], Dip et al. (2019), and Parfentiev [35], with 196, 170, and 58 patients, respectively. The largest study was conducted by Rudin et al. [29], which included 210 patients.

It's important to note that this study acknowledges some limitations. First, the design did not involve random assignment of patients to groups, which can introduce bias. Second, the study involved a relatively small number of participants. Third, the large camera used limited the ability to see parathyroid glands in certain areas. A smaller, handheld camera might address this issue.

However, to draw more definitive conclusions, additional studies such as randomized controlled trials and meta‐analyzes are necessary. Large‐scale prospective multicenter studies are necessary across diverse patient populations undergoing thyroid and parathyroid surgery to confirm the clinical usefulness of these promising detection techniques [56].

## 5 Conclusions

ICG angiography is a simple and quick procedure that does not prolong surgery. The process of preparing and injecting the fluorescent dye is straightforward, and the maneuvers required are not complex or challenging.

Surgeons primarily use ICG to improve visualization of the parathyroid glands (PGs) during surgery. ICG has been shown to be a valuable tool that helps surgeons locate and assess these glands more effectively compared to traditional imaging techniques alone. However, some studies have reported conflicting results, which may be due to the small number of patients involved in these studies and variations in how researchers measured the effectiveness of ICG.

## Author Contributions

All authors participated in data collection, interpretation, drafting, and revision of the manuscript, and approved the final version for publication.

## Ethics Statement

The study adhered to ethical guidelines and regulations. All participants provided written informed consent, and the research was approved by the ethics committee at Tehran University of Medical Sciences. This included the use of near‐infrared fluorescence imaging with ICG.

## Consent

The patients gave their written permission for their cases to be included in this report.

## Conflicts of Interest

Dr. Maziar Motiee‐Langroudi, Dr. Athena Farahzadi, Dr. Mohammad Shirkhoda, Prof. Habibollah Mahmoodzadeh, Prof. Ramesh Omranipour, Dr. Amirmohsen Jalayifar, and Prof. Iraj Harirchi have no conflicts of interest to reveal.

## Supporting information


**Video S1.** Intraoperative view using near‐infrared fluorescence angiography with indocyanine green (ICG) demonstrating two well‐perfused parathyroid glands (highlighted) after elevation of the right thyroid lobe.

## Data Availability

The data that support the findings of this study are available on request from the corresponding author. The data are not publicly available due to privacy or ethical restrictions.
